# Positive Effect of Kinesiotape on 1 km Run Performance in University-Level Males: A Cross-Sectional Study

**DOI:** 10.3390/jfmk7020032

**Published:** 2022-04-12

**Authors:** Peter Bartík, Peter Šagát

**Affiliations:** Health and Physical Education Department, Prince Sultan University Riyadh, Riyadh 12435, Saudi Arabia; sagat@psu.edu.sa

**Keywords:** kinesiotape, performance, run

## Abstract

**Introduction:** The kinesiotape (KT) method is used to exert a positive effect on muscular, nervous, and organ systems, recognizing the importance of muscle movement. It is widely applied in runners for performance enhancement. However, there is no scientific background to use it as a running speed modulator. **Objectives:** The purpose of this study was to verify the KT effect on running performance in university-level students while speed is considered. The 1 km run and 40 m shuttle run were investigated. Participants were highly motivated to run as fast as possible since the research was part of the graded fitness test. Students wanted to perform as well as possible to get good marks. **Methods:** A total of 150 students aged 19.93 ± 0.85 with BMIs of 26.93 ± 0.98 were randomly distributed to the experimental (EG), placebo (PG), and control group (CG). In the EG, 50 students were measured pre-test (no KT) and post-test (KT applied). In the PG, 50 students were measured the same way using the placebo application post-test. In the CG, 50 students were measured without any intervention pre-test or post-test. The application area was the triceps surae muscle bilaterally with KT tension of 75%. The Kruskal–Wallis test and repeated measures ANOVA were used for analysis with a 0.05 level of significance. **Results:** A statistically significant group effect was reported in the EG (<0.05) in the 1 km run discipline. The time results obtained were significantly shorter than in the PG and the CG. There was no statistically significant difference (0.717) reported in the 40 m shuttle run discipline between the groups. **Conclusions:** Applying KT with a tension of 75% on the triceps surae muscle bilaterally might be useful to increase the performance of medium or longer distance runs but may not be effective in improving sprinting ability. We recommend applying the KT in the overall muscle and tendon area with a tension of 75% if there is a focus on performance enhancement.

## 1. Introduction

Kinesiotape, Kineziotape, Kinesiotaping, or Kinesthetic tape are just a few of the common terms that are used for a specific diverse method that uses special elastic tape to treat and improve the muscular, nervous, and organ systems [1]. The primary tool of this unique method is an elastic, multicolored tape with an acrylic adhesive component that is applied on the skin. The method itself is related to kinesiology science, recognizing the importance of muscle movement during daily life. Kinesiotape (KT) is composed of narrowly interlaced, high-strength elastic cotton fibers that are highly resistant to mechanical load and water. It contains no drug and no other active substance—all the benefits described are based on its elastic qualities [2]. These elastic attributes should also play a supportive role in reducing the biomechanical load of the affected muscle [3]. The elasticity of the KT fibers is present only in the longitudinal direction, taking into account the anatomy of the muscle fibers. To stretch it transversally is only possible in a very limited range. The system of KT application cannot be changed with the classic adhesive rigid tape technique which is used for joint immobilization and support. However, we can see an effective combination of these two techniques in sports [4]. KT’s visibility has grown even further after the 2008 Olympic Games when it was widely used by athletes, but despite the increasing popularity, there is still a necessity for scientific evidence to fully support its benefits [5]. No publications, articles, or the author himself mention any side effects, but it is not used in the case of open wounds, irritated skin, on sacral body parts, and in the case of allergy to the acrylic adhesive component. The important factor is the length of the application, which is not limited by space and environment as other physiotherapeutic techniques. The effect is continuous for 24 h until the tape is removed manually from the body. The maximum duration of application is 4–5 days as KT gradually loses its elasticity. Moore [6] summarized the mechanical effects of KT into these points:

1. Support for muscle function;
2. Improving blood and lymph circulation through muscle and subcutaneous movement and accelerating metabolism in the area;
3. Reducing pain by raising the subcutaneous tissue layer and reducing the chemoreceptor irritation;
4. Reducing pain by increased sensory stimulation and change in pain perception—gate control theory;
5. Increasing proprioception by influencing skin mechanoreceptors.

Several studies have reported that KT applied on the skin can improve afferent signals to the central nervous system and thereby improve proprioception [2,7]. Others have indicated that KT may have a short-term positive effect on muscle activation and even range of motion [8]. The continuous tension applied to the skin should activate the mechanoreceptors, which can then stimulate modulation mechanisms within the central nervous system and subsequently enhance muscular excitability [9].

Studies dedicated to measuring the KT effect on ankle and calf function or performance, predominantly use an ipsilateral movement model. The often-applied test is jump. The following authors used this model in their study: [10,11,12,13,14]. From the physiological point of view, the ipsilateral model is less natural for the human body. A more natural and physiological variant of testing is contralateral movement. The contralateral model is the only method that can assess both the central and peripheral mechanisms associated with proprioception [15]. In our experiment, we have decided to test the KT effect on running as an ideal possibility of physiological contralateral movement. For the application, we chose the area of the triceps surae and Achilles tendon bilaterally. The triceps surae is unique in that it combines the functions of two muscles, soleus and gastrocnemius, through a shared aponeurosis in series with a common tendon [16]. The Achilles tendon is the strongest and one of the largest in the human body [17]. This architectural arrangement allows the unit to store energy and maximize the motor performance and metabolic efficiency of the lower limb [18]. The distribution of fiber type within the triceps surae suggests that gastrocnemius has a greater role in power generation (type II a/b) and that soleus is designed for endurance (type I). The power role of gastrocnemius is further supported by its fibers being longer, shortening faster, and working over a larger range of movement compared to soleus [19]. Soleus is the prime muscle involved in forward propulsion and acceleration [20], whereas gastrocnemius allows power to be transferred through the legs and initiates leg swing [21]. The power-transferring function of gastrocnemius is particularly important in explosive sports movements, such as sprinting and jumping [22]. The gastrocnemius muscle is located superficially unlike the soleus, and the KT effect might be more significant in this matter. KT application might support muscle performance by the activation technique and be more significant in the short run than the longer one, where speed, muscle activation, and propulsive energy are inevitable, and the direct effect of KT on muscle fibers seems to enhance exactly these muscular abilities.

### Objectives

The purpose of this study was to verify the KT effect on running performance in university-level students while speed is considered. The 1 km run and 40 m shuttle run were investigated.

## 2. Methods and Materials

A total of 150 students of Prince Sultan University aged 19.93 ± 0.85 and with BMIs of 26.93 ± 0.98 agreed to participate in the research. All participants were male recreational runners, running distances up to 5 km twice a week. They were participating in running and running-related exercises during their regular classes. Detailed subject characteristics are reported in Table 1. The inclusive criteria to keep homogeneity and the same initial parameters were the following. Participants were the ages of 19–21. Body mass index of 25–30 was chosen as it best represented the sample of recreational runners at Prince Sultan University. All students had appropriate sleep (at least 7 h) before the test and did it at the same time in the morning. All students had a light breakfast. Participants signed informed consent forms and were free to step out from the research at any time. As a test unit, we measured the performance of the 1000 m run and 40 m shuttle run (10 m track x 4). The 1000 m run and 40 m shuttle run are the standardized evaluation methods used in the assessment of PE students worldwide. Both are part of the International Physical Fitness Test (IPFT) battery [23]. The unit consists of two measurement periods, pre-test and post-test. For the accurate measurement of the 40 m shuttle run, we used the photo-cell machine (Brower TCi System, Brower Timing Systems, Draper, UT, USA). The 1 km run was performed on the treadmill where a distance of 1000 m was pre-set in the program and students were responsible for selecting and managing their speed. The Cybex 625T (Life Fitness, Rosemont, IL, USA) treadmill was used in our research. The treadmill was preferred to outdoor running due to the weather conditions in Riyadh, including high levels of dust pollution, aridity, temperature, and also the quality of the running surface. Moreover, most students at PSU prefer to and are used to running in indoor facilities. The treadmill speed was set up based on individual preferences, and they started the treadmill acceleration with 0 km. No other devices, such as heart rate monitors, were used in the measurements. The motivation factor was that the mentioned tests are standardized at the university, and the final grade is affected by the results of both. All participants were motivated to perform at their best during both the pre-test and post-test. The correct technique was familiar to all probands as they train for the mentioned disciplines at Prince Sultan University regularly. The subjects are amateur runners training regularly two times a week. There were 10 training sessions (within one month) conducted prior to the testing events to support the technique of the shuttle run and 1 km run. Everybody performed a 10 min warm-up before. All filled out a medical questionnaire to clarify their good medical condition. The pause between the tests was one day.

**Figure 1:**
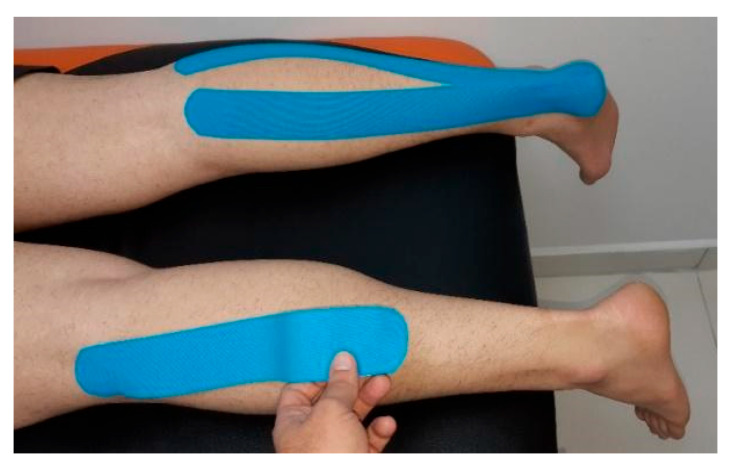
KT application on triceps surae.

**Figure 2:**
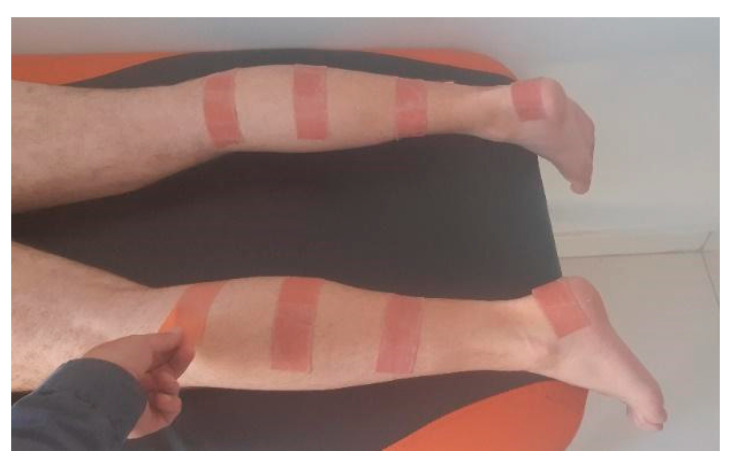
Placebo tape application.

A total of 150 participants who met the inclusive criteria were randomly assigned to the experimental (EG), placebo (PG), or control group (CG). In the CG, we measured the performance without KT in the pre-test and the post-test as well, following the PG with measurement of performance without any tape in pre-test and with placebo tape applied in post-test. In the EG, we measured the performance without KT in the pre-test and with KT applied in post-test. Each group had 50 individuals. Probands who suffered from any kind of physical problem, such as low back pain, post-operation condition, or any other discomfort that could affect the results of tests, were excluded from the research. All EG probands were asked to shave both of their calves. Twenty-four hours prior to the tests, KT was applied on bilateral triceps surae while in prone position (Figure 1). We used the technique for muscular facilitation. KT (Mueller) was applied from triceps surae origin (area of medial and lateral tibial condyles and popliteal fossa posteriorly) to its insertion (distal calcaneus). Two 5 cm wide KT stripes were applied on disinfected skin, one for each muscular head. The tension used was 75%, as recommended by Kase et al. (2013). The 75% tension was reached by extending the tape from its neutral position to 100% and releasing the maximal tension by one quarter afterward. The KT was applied by a professional experienced specialist in kinesiotaping and physiotherapy. Placebo tape (Endura rigid tape) was applied prior to post-test in PG on the bilateral calf area with no tension and no anatomical direction (Figure 2) The application of the rigid tape in the placebo group was done randomly, simulating naive application techniques used by many athletes having no or little idea about anatomical structures, physiotherapeutic knowledge, or professional guidance in the field of kinesiotaping. There was not any input information provided to participants prior to the tests about the effective/noneffective impact of KT or the placebo.

### 2.1. Statistical Analysis

Statistical analysis was performed by IBM SPSS statistical software version 25.0. The Kruskal–Wallis test and Repeated Measures ANOVA were used for analysis with the 0.05 level of significance used for each discipline with the appropriate post hoc tests. The normal distribution of data was analyzed by the Kolmogorov–Smirnov and Shapiro–Wilk tests. The assumption of sphericity was tested using Mauchly’s test. Statistical significance was set at the level of *p* < 0.05. Descriptive characteristics of the subjects are presented in Table 1. The mean and standard deviation results for each of the disciplines and particular groups are presented in Table 2.

### 2.1.1. 1 km Run

Since the normal distribution was not confirmed in the EG and PG, we used a non-parametric Kruskal-Wallis (K-W) test to compare the differences between the groups. The K-W test confirmed statistically significant differences between the groups. The comparison of the groups is reported by post-hoc tests in Table 3.

The significant differences were shown between the two comparisons with the EG. The EG mean rank is significantly lower than in both PG and CG. The *p*-value is <0.001 for both tests.

### 2.1.2. 40 m Shuttle Run

The normal distribution was confirmed; thus, the parametric ANOVA test to compare the differences was used. The results are presented in Table 4.

Based on the result of the ANOVA test (*p*-value 0.717), the time difference of repeated measurement is similar in all groups—experimental, control, and placebo.

## 3. Results

**Figure 3:**
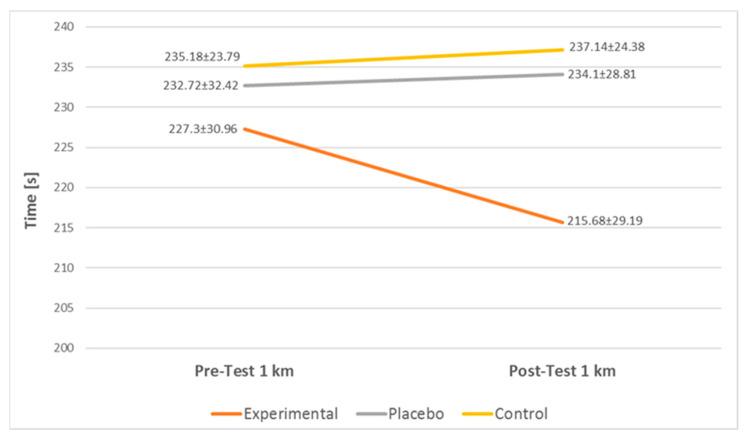
1 km run results.

**Figure 4:**
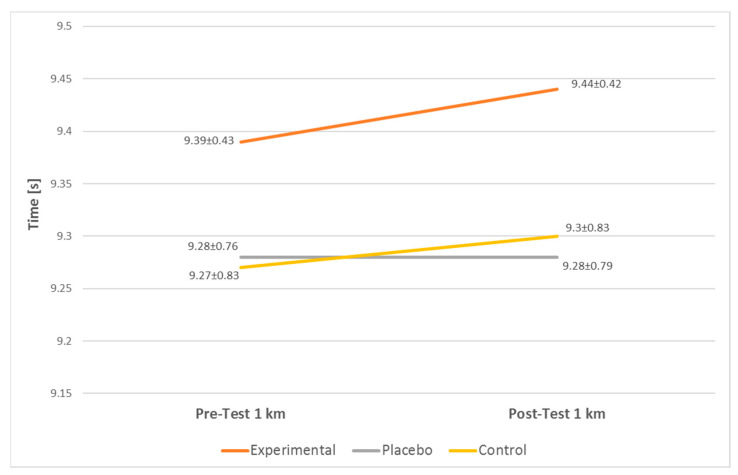
40 m shuttle run results.

The results of the present study show a significant difference in the EG 1 km run discipline but do not show any significant difference in the other two groups. The 1 km run results are illustrated in Figure 3. There was no statistically significant difference reported in the 40 m shuttle run discipline between the groups. The 40 m run results are illustrated in Figure 4.

## 4. Discussion

In our study, the KT effect for a shorter 40 m shuttle sprint was not significant, while there was a statistically significant difference in the 1 km run obtained. We attributed this outcome to the impact of the KT. All 150 male students were assigned randomly to three research groups, EG, PG, and CG. Each group reported different mean values of the initial pre-test in both disciplines, referring to various performance categories of the participants. However, only a 1 km run of the EG reported a significant improvement in the post-test. The KT effect on activation and enhanced muscular fiber reactibility still divides scientists into two groups and the results are not entirely clear. In recent years, a number of studies have been revealed, as well as meta-analyses that support but also oppose this theory. Saavedra-Hernandez et al. [24] consider the KT effect on muscle fiber activation controversial, despite the results of several other studies. A composite of authors claims that the evidence does not support the use of KT in practice, and many studies are based on overestimated results. They also point to gaps in the literature describing the technique of application [25,26,27]. Our experience of the KT effect on performance is different. The previous assumption that the effect will be more significant in a short track run where speed and rebound are important appears improbable. Similarly, the KT effect was not significant in the 20 m sprint in healthy rugby players, where KT was applied on the gluteal area [28]. Based on our study outcome, we recommend using KT for a longer distance run (compared to short sprints or shuttle run); in our case, it was the 1 km run. The KT effect might decrease stabilization and muscle activation demands and makes running more economic. Thus far, 17 studies have reported that exoskeletons improve natural human walking and running economy [29]. Several focused on the calf and ankle joint with positive effects on running and walking economy supporting the idea of our KT application place [30,31,32].

Freedman et al. [33] reported a short-term moderate improvement in muscle performance and a pain reduction in the single leg hop test in patients with patellofemoral pain syndrome. There was a patellar-taping technique performed. Akbaba et al. [34] compared the KT effect on pain and function in patients who received a verbal explanation of its positive effect with the group to which KT was applied without input information. The effect on pain and function was insignificantly higher in patients with positive input information, but the difference was not clinically meaningful. In our study, the CG, PG, and EG participants were all motivated the same way for maximum performance for the pre-test and post-test. All probands did not receive any information about KT or the placebo potential impact prior to the tests. Aguiar et al. [35] investigated the effect on dominant handgrip strength at 48 h and 72 h after KT application. The test did not reveal a significant later effect in the grip strength. The muscle inhibition application technique induced muscular strength reduction by 8.40%. No statistically significant differences in maximal muscle grip strength in healthy subjects before and after KT application were found by Chang et al. [2]. Saracoglu et al. [36] investigated the impact of KT on pain, range of motion, and muscle strength in 135 patients diagnosed with subacromial impingement syndrome. Regarding the research results, the authors reported that KT can be the optimal method to improve the condition, especially in the initial phase. The investigation of the KT effect on chronic ankle instability did not indicate that muscle activity facilitation or inhibition was present [37]. In our study, the KT effect for a shorter 40 m track sprint was not significant as well. The effect was notable only on a longer 1 km run. Kaya-Kara et al. [38] published the study about the effect of taping in children with cerebral palsy. By specific functional tests, they noted significant differences in performance as muscle efficiency increased after KT application. The important question about the effect on muscle performance is whether the intensity on a healthy muscle corresponds to the effect on a weakened muscle. We presume that the magnitude of the effect will be more significant in a weak muscle, although this theory is not confirmed and should certainly be the subject of a future study. Centner and Salinas [39] measured the KT impact on the concentric muscle strength of the rectus femoris and the tibialis anterior muscle in healthy individuals. They did not ascertain any significant difference in the research before and after the use of KT. A similar result was observed by Oliveira et al. [40] who explored the eccentric and concentric isokinetic muscle contraction of vastus lateralis. According to the authors, KT did not modulate the neuromuscular performance of the quadriceps femoris. As a possible explanation, they reported inadequate tactile stimulus generated by tape, which was not sufficient to induce muscle contraction. A different outcome of the KT impact was reported by Vithoulk et al. [41] in measuring m. quadriceps femoris activation in healthy women. The results revealed a significant statistical increase in eccentric isokinetic activation during the peak torque test. The difference in the result of concentric and eccentric activation is attributed to the different length of the individual motions tests. A possible explanation is that if the KT were a muscle tone regulator, its effect would be more recognizable with longer muscle activation. We identify with this possible explanation; the length of the KT application test and the muscle activation length as well are important in the matter of the final outcome. The KT effect might be more significant in this case, which was confirmed in our study as well.

We presume that the various areas of KT applications may play an important role in the different results of the mentioned studies. Some authors only applied it to individual quadriceps muscles. In the case of Centner and Salinas [39], it was only the rectus femoris muscle. Oliveira et al. [40] measured the KT effect on the vastus lateralis muscle itself. Vithoulk et al. [41] with a positive result measured the facilitation of the whole quadriceps femoris, and KT was applied on the rectus femoris, vastus medialis, and vastus lateralis. In our study, we used two stripes of classic 5 cm wide KT to cover all the triceps surae area. Presumably, this fact causes increased tactile stimulus of muscle contraction and segmental stability, and it is crucial for the study’s outcome. Furthermore, it should also be noted that the method of KT application is not the same for individual research. Its effect is influenced by several factors. In addition to the above-mentioned application area, the direction and intensity of KT tension are equally important. In the case of tension direction, researchers mostly respect the principles of KT from the author of the methodology. When facilitating muscle, they apply a pull from origin to insertion in the direction of muscle fiber activation respecting the anatomy. However, in particular studies, we found different qualities of KT tension which might be crucial to the results. The tension recommended by the author of the methodology, ideal for facilitating muscle fibers, is 75%. It is achieved by pulling the KT by 100% after taking it off from the cover paper and reducing by one quarter of the length reached [1,42]. Song et al. [43] applied a 20% tension of KT in their research. A similar range was also used by Aguiar et al. [35] in measuring the force of a grip. KT was applied with a tension of 10–15%. Haerle and Zwiebel [44] used 25–50% tension for muscle activity facilitation. Aguilar-Ferrándiz et al. [8] reported 15–50%. Both Davis [45] and Oliveira et al. [40] investigated the KT effect on performance with the tension of 40–60%. Cai et al. [46], as well as Aghapour et al. [47] used the above-mentioned value of 75% tension in their studies. Our research team used the same tension of 75%. We believe that the inconsistency of individual application methods has a significant impact on the research results.

Our preliminary hypothesis that KT may positively affect short track run performance due to its effect on propulsive energy was not confirmed. Vice versa, the effect was more significant on a 1 km long track. In accordance with the positive impact on a longer 1 km track, we assume that except for the muscle activation element, the segment stabilization played a role in the final result as well. We must realize that 75% KT tension with origin from lateral and medial tibial condyle and insertion below the ankle on the distal calcaneus may provide increased stability for the ankle joint. This intense ankle stability may have an impact on the 1 km track due to the longer time and distance, and its presence was more evident than in a shorter 40 m track. We presume that the effect in a longer distance run decreases the physical demands on stabilization and muscle activation and supports the economy of running. However, this will be the objective of future studies. The limitation of the study was the usage of KT on the triceps surae complex only. The result of running performance might be more significant in the case of wider application (e.g., quadriceps femoris muscle). The limitation was also the different performance abilities of particular participants. The subjects of BMI category A (BMI 18.5–25) might show different results.

## 5. Conclusions

Applying KT with a tension of 75% on the triceps surae muscle bilaterally might be useful to increase the performance of medium or longer distance runs but may not be effective to improve the sprinting ability. We recommend applying the KT to the overall muscle and tendon area with a tension of 75% if there is a focus on performance enhancement.

## Figures and Tables

**Table 1:** Subjects Characteristics.

Group	N	AGE		BMI	
		Mean	Std. Dev.	Mean	Std. Dev.
Experimental	50	19.84	0.77	26.59	1.12
Placebo	50	20.02	0.87	27.13	0.77
Control	50	19.94	0.91	27.05	0.96
**Summary**	**150**	**19.93**	**0.85**	**26.93**	**0.98**

**Table 2:** Results of 1 km and 40 m shuttle run in seconds.

Group	Pre-Test 1 km		Post-Test 1 km		Pre-Test 40 m		Post-Test 40 m	
	Mean	St. Dev.	Mean	St. Dev.	Mean	St. Dev.	Mean	St. Dev.
Experimental	227.30	30.96	215.68	29.19	9.39	0.43	9.44	0.42
Placebo	232.72	32.42	234.10	28.81	9.28	0.76	9.28	0.79
Control	235.18	23.79	237.14	24.38	9.27	0.83	9.30	0.83

**Table 3:** Post-hoc test 1 km.

	Group	N	Mean Rank	Sum of Ranks
Difference 1 km	Experimental	50	33.15	1657.50
	Control	50	67.85	3392.50
	Total	100		
Difference 1 km	Experimental	50	37.29	1864.50
	placebo	50	63.71	3185.50
	Total	100		
Test Statistics-Grouping Variable: Group				
			Diiference 1 km	
Mann–Whitney U			382.500	
Wilcoxon W			1657.500	
Z			−5.984	
Asymp. Sig. (2-tailed)			0.000	
Mann–Whitney U			589.500	
Wilcoxon W			1864.500	
Z			−4.557	
Asymp. Sig. (2-tailed)			0.000	

**Table 4:** ANOVA—40 m shuttle run.

	Sum of Squares	df	Mean Square	F	Sig.
Between Groups	0.082	2	0.041	0.334	0.717
Within Groups	18.095	147	0.123		
Total	18.177	149			

## Data Availability

This study was conducted under the project “Philosophical and ethical aspects of sport”, project code: NP-KSV-ET-01-2021-12/SP.

## References

[1] Kase K., Wallis J., Kase T. (2013). Clinical Therapeutic Applications of the Kinesio Taping Method.

[2] Chang H.-Y., Chou K.-Y., Lin J.-J., Lin C.-F., Wang C.-H. (2010). Immediate effect of forearm Kinesio taping on maximal grip strength and force sense in healthy collegiate athletes. Phys. Ther. Sport.

[3] Bassett K.T., Lingman S.A., Ellis R.F. (2010). The use and treatment efficacy of kinaesthetic taping for musculoskeletal conditions: A systematic review. N. Z. J. Physiother..

[4] Kumbrink B. (2014). K-Taping.

[5] Williams S., Whatman C., Hume P., Sheerin K. (2012). Kinesio Taping in Treatment and Prevention of Sports Injuries. Sports Med..

[6] Moore R. (2012). What is the current evidence for the use of kinesio tape? A literature review. SportEX Dyn. J..

[7] Tunay V.B., Akyuz A., Onal-Aykar S., Usgu G. (2008). Comparison of the instant effects of kinesio and McConnell patellar taping on performance in patellofemoral pain syndrome. Turk. J. Physiother. Rehabil..

[8] Aguilar-Ferrándiz M.E., Moreno-Lorenzo C., Matarán-Peñarrocha G.-A., García-Muro F., García-Ríos M.D.C., Castro-Sánchez A.M. (2014). Effect of a Mixed Kinesio Taping–Compression Technique on Quality of Life and Clinical and Gait Parameters in Postmenopausal Women with Chronic Venous Insufficiency: Double-Blinded, Randomized Controlled Trial. Arch. Phys. Med. Rehabil..

[9] Gómez-Soriano J., Abián-Vicén J., Aparicio-García C., Ruiz-Lázaro P., Simon-Martinez C., Bravo-Esteban E., Fernández-Rodríguez J.M. (2013). The effects of Kinesio taping on muscle tone in healthy subjects: A double-blind, placebo-controlled crossover trial. Man. Ther..

[10] Huang C.-Y., Hsieh T.-H., Lu S.-C., Su F.-C. (2011). Effect of the Kinesio tape to muscle activity and vertical jump performance in healthy inactive people. Biomed. Eng. Online.

[11] Nunes G.S., de Noronha M., Cunha H.S., Ruschel C., Borges N.G. (2013). Effect of Kinesio Taping on Jumping and Balance in Athletes: A crossover randomized controlled trial. J. Strength Cond. Res..

[12] Vinken P. (2015). Short-term effects of elastic taping on gymnast’s jumping performance. Sci. Gymnast. J..

[13] Glória I.P.D.S., Herpich C.M., Serenza F., Politti F., Gonzalez T.D.O., Gomes C.A.F.D.P., Biasotto-Gonzalez D.A. (2020). Is Kinesio taping better than placebo taping for improving performance during unilateral vertical jump and hop tests? Protocol study for a randomized, placebo-controlled, double-blind, clinical trial. Man. Ther. Posturology Rehabil. J..

[14] Tamura K., Resnick P.B., Hamelin B.P., Oba Y., Hetzler R.K., Stickley C.D. (2020). The effect of Kinesio-tape^®^ on pain and vertical jump performance in active individuals with patellar tendinopathy. J. Bodyw. Mov. Ther..

[15] Docherty C.L., Gansneder B.M., Arnold B.L., Hurwitz S.R. (2006). Development and reliability of the ankle instability instrument. J. Athl. Train..

[16] Blitz N.M., Eliot D.J. (2008). Anatomical Aspects of the Gastrocnemius Aponeurosis and its Muscular Bound Portion: A Cadaveric Study—Part II. J. Foot Ankle Surg..

[17] Kannus R., Jòzsa L., Renström R., Järvtoen M., Kvist M., Lento M., Oja P., Vuorl I. (1992). The effects of training, immobilization and remobilization on musculoskeletal tissue. Scand. J. Med. Sci. Sports.

[18] Benjamin M., Kaiser E., Milz S. (2008). Structure-function relationships in tendons: A review. J. Anat..

[19] Arampatzis A., De Monte G., Karamanidis K., Morey-Klapsing G., Stafylidis S., Brüggemann G.-P. (2006). Influence of the muscle-tendon unit’s mechanical and morphological properties on running economy. J. Exp. Biol..

[20] McGowan C., Kram R., Neptune R. (2009). Modulation of leg muscle function in response to altered demand for body support and forward propulsion during walking. J. Biomech..

[21] Sasaki K., Neptune R.R. (2006). Differences in muscle function during walking and running at the same speed. J. Biomech..

[22] Jacobs R., Bobbert M.F., Schenau G.J.V.I. (1996). Mechanical output from individual muscles during explosive leg extensions: The role of biarticular muscles. J. Biomech..

[23] Rosandich T.P. (2003). The international physical fitness test manual. Sport J..

[24] Saavedra-Hernández M., Castro-Sánchez A.M., Arroyo-Morales M., Cleland J.A., Lara-Palomo I.C., Fernández-De-Las-Peñas C. (2012). Short-Term Effects of Kinesio Taping Versus Cervical Thrust Manipulation in Patients with Mechanical Neck Pain: A Randomized Clinical Trial. J. Orthop. Sports Phys. Ther..

[25] González-Iglesias J., Fernández-De-Las-Peñas C., Cleland J., Huijbregts P., Gutiérrez-Vega M.D.R. (2009). Short-Term Effects of Cervical Kinesio Taping on Pain and Cervical Range of Motion in Patients with Acute Whiplash Injury: A Randomized Clinical Trial. J. Orthop. Sports Phys. Ther..

[26] Mostafavifar M., Wertz J., Borchers J. (2012). A Systematic Review of the Effectiveness of Kinesio Taping for Musculoskeletal Injury. Physician Sportsmed..

[27] Parreira P.D.C.S., Costa L.D.C.M., Junior L.C.H., Lopes A., Costa L. (2014). Current evidence does not support the use of Kinesio Taping in clinical practice: A systematic review. J. Physiother..

[28] Strutzenberger G., Moore J., Griffiths H., Schwameder H., Irwin G. (2015). Effects of gluteal kinesio-taping on performance with respect to fatigue in rugby players. Eur. J. Sport Sci..

[29] Sawicki G.S., Beck O.N., Kang I., Young A.J. (2020). The exoskeleton expansion: Improving walking and running economy. J. Neuroeng. Rehabil..

[30] Malcolm P., Lee S., Crea S., Siviy C., Saucedo F., Galiana I., Panizzolo F.A., Holt K.G., Walsh C.J. (2017). Varying negative work assistance at the ankle with a soft exosuit during loaded walking. J. Neuroeng. Rehabil..

[31] Mooney L.M., Herr H.M. (2016). Biomechanical walking mechanisms underlying the metabolic reduction caused by an autonomous exoskeleton. J. Neuroeng. Rehabil..

[32] Collins S.H., Wiggin M.B., Sawicki G.S. (2015). Reducing the energy cost of human walking using an unpowered exoskeleton. Nature.

[33] Freedman S.R., Brody L.T., Rosenthal M., Wise J.C. (2014). Short-Term Effects of Patellar Kinesio Taping on Pain and Hop Function in Patients with Patellofemoral Pain Syndrome. Sports Health Multidiscip. Approach.

[34] Akbaba Y.A., Mutlu E.K., Altun S., Gümüşoğlu G., Çelik D. (2017). The effects of Kinesio Tape application with different verbal input given to with patients with rotator cuff tear. Orthop. J. Sports Med..

[35] Aguiar R.S.N.D.A., Boschi S.R.M.D.S., Lazzareschi L., da Silva A.P., Scardovelli T.A., Filoni E., Manrique A.L., Frère A.F. (2018). The late effect of Kinesio Taping^®^ on handgrip strength. J. Bodyw. Mov. Ther..

[36] Saracoglu I., Emuk Y., Taspinar F. (2017). Does taping in addition to physiotherapy improve the outcomes in subacromial impingement syndrome? A systematic review. Physiother. Theory Pract..

[37] Kodesh E., Dar G. (2015). The effect of kinesiotape on dynamic balance following muscle fatigue in individuals with chronic ankle instability. Res. Sports Med. Int. J..

[38] Kaya Kara O., Atasavun Uysal S., Turker D., Karayazgan S., Gunel M.K., Baltaci G. (2014). The effects of Kinesio Taping on body functions and activity in unilateral spastic cerebral palsy: A single-blind randomized controlled trial. Dev. Med. Child Neurol. J..

[39] Centner K., Salinas A. (2014). The Effect of Kinesio^®^ Tape on Concentric Force Production of the Rectus Femoris and Tibialis Anterior in Healthy, Un-Injured Individuals. Ph.D. Thesis.

[40] Oliveira A.K., Borges D.T., Lins C.A., Cavalcanti R.L., Macedo L.B., Brasileiro J.S. (2016). Immediate effects of Kinesio Taping^®^ on neuromuscular performance of quadriceps and balance in individuals submitted to anterior cruciate ligament reconstruction: A randomized clinical trial. J. Sci. Med. Sport.

[41] Vithoulka I., Beneka A., Malliou P., Aggelousis N., Karatsolis K., Diamantopoulos K. (2010). The effects of Kinesio-Taping^®^ on quadriceps strength during isokinetic exercise in healthy non athlete women. Isokinet. Exerc. Sci..

[42] Kumbrink B. (2016). K-Taping in Pediatrics—Basics Techniques Indications.

[43] Song C.-Y., Huang H.-Y., Chen S.-C., Lin J.-J., Chang A.H. (2015). Effects of femoral rotational taping on pain, lower extremity kinematics, and muscle activation in female patients with patellofemoral pain. J. Sci. Med. Sport.

[44] Haerle M.K., Zwiebel R.J. (2017). Does the Direction of Application of Kinesio^®^Tape Have an Effect on Time to Peak Muscle Torque of the Concentric Contraction of the Quadriceps Muscle in Healthy Young Adults?. Ph.D. Thesis.

[45] Davis R.F. (2013). The Accute Effects of Kinesiotape on Throwing Velocity in Collegiate Baseball Athletes. Ph.D. Thesis.

[46] Cai C., Au I.P., An W.W., Cheung R.T.H. (2015). Facilitatory and inhibitory effects of Kinesio tape: Fact or fad?. J. Sci. Med. Sport.

[47] Aghapour E., Kamali F., Sinaei E. (2017). Effects of Kinesio Taping^®^ on knee function and pain in athletes with patellofemoral pain syndrome. J. Bodyw. Mov. Ther..

